# Open defecation-free slippage and its associated factors in Ethiopia: a systematic review

**DOI:** 10.1186/s13643-020-01511-6

**Published:** 2020-11-03

**Authors:** Thomas Ayalew Abebe, Gudina Terefe Tucho

**Affiliations:** Department of Environmental Health Sciences and Technology, Jimma University, P.O. Box 387, Jimma, Ethiopia

**Keywords:** Defecation, Hygiene, Sanitation, Ethiopia

## Abstract

**Background:**

Recent studies have shown an increase in open defecation and slippage of open defecation-free certified villages in Ethiopia, despite significant progress the country made on sanitation programs. Hence, realizing of existing facts, this study was conducted aiming at a critical review of available literature and to provide consolidated data showing the level of slippage and its associated factors in Ethiopia.

**Methods:**

Systematic literature searches were performed from four international databases. The search involved articles published from December 1, 2013, up to June 4, 2019. The Cochran’s *Q* and *I*^2^ test statistics were used to check heterogeneity among the studies. To negotiate heterogeneity from qualitative data, we used a mixed-method approach. The researchers also conducted a publication bias assessment and sensitivity analysis. A random effect meta-analysis was employed to determine the pooled estimates of open defecation free slippage rate with a 95% confidence interval (CI). The data analysis was performed using the CMA V.3 software program.

**Result:**

After screening 1382 studies, 12 studies were finally included in this systematic review. The estimated pooled rate of open defecation-free slippage in Ethiopia was 15.9% (95% CI 12.9–19.4%). The main contributing factors for open defecation-free slippage were lack of technical support, financial constraints, low-quality building materials, improper program implementation, and lack of sanitation marketing.

**Conclusion:**

It was estimated that 1 out of 6 Ethiopian households engaged in open defecation after they have certified open defecation-free status, implying the low possibility of achieving sustainable development goals of 2030, which aims to ensure sanitation for all. Therefore, the government of Ethiopia and donors should better give special attention to the following options: (1) awareness for open defecation-free slippage, (2) launch a post-open defecation-free program, and (3) encourage research on pro-poor sustainable sanitation technologies.

**Supplementary Information:**

The online version contains supplementary material available at 10.1186/s13643-020-01511-6.

## Background

Globally, around 0.9 billion people practice open field defecation. Recent reports showed a drop in the number of individuals practicing open defecation in many regions of the world. However, sub-Saharan African countries increased the number of people defecating in the open field from 204 million to 220 million [1].

The reasons for increasing open defecation in Sub-Saharan Africa are high population growth and the slippage of open defecation-free (ODF)-certified communities, which refers to community member failure to keep fulfilling all open defecation free criteria. The investigation in African countries confirmed that the ODF slippage rate on the continent is 10–13% per year. Here, researchers defined the term ODF slippage based on sub-optimal latrine utilization and open field defecation [2].

In Ethiopia, the sanitation program has given special attention since 1995 after the government incorporated public health in the National Constitution. Subsequently, the Ministry of Health developed the National Hygiene and Sanitation Strategy and National Hygiene and On-Site Sanitation Protocol in 2005 and 2006 consecutively [3]. In 2006, an Irish NGO “VITA” introduced a sanitation tool called Community-Led Total Sanitation and Hygiene (CLTSH) program [4].

Community-Led Total Sanitation and Hygiene program is a better approach toward the reduction of open defecation practice and the achievement of the desired sanitation program. From 2011 to 2015, all Ethiopian administrative regions implemented a Community-Led Total Sanitation and Hygiene program covering more than 80% of the districts. According to the Ethiopian Ministry of Health report in 2015, 4657 kebeles (administrative villages) declared for open defecation-free status [3, 5].

**Fig. 1:**
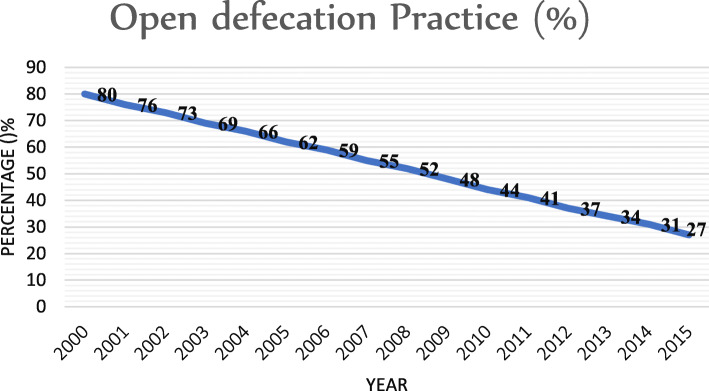
Open defecation status in Ethiopia from 2000 to 2015 (source: SH.STA.ODFC.ZS)

Similarly, reports from the Ethiopian Demographic Health Survey indicated a decreasing trend of open defecation: 81.9% in 2000, 61.9% in 2005, 38.3% in 2011, and 32.9% in 2016 [6–9]. The WHO and UNICEF Joint Monitoring Program data also showed that Ethiopia tremendously decreased open defecation between the years 2000 and 2015 (Fig. 1) [1]. However, a recent report showed that the practice of open defecation in Ethiopia is increasing in the same way it was rising in sub-Saharan African countries [3, 10], and approximately 35.6% of the population engaged in open defecation. This means that Ethiopia is off-tracking from the achievement of the Sustainable Development Goal (SDG), where half of the population still relies on unimproved sanitation facilities and 50% latrine utilization [11].

The construction of more latrines does not ensure a drop in disease among people, especially children. However, open defecation-free status will reduce the occurrence of associated illnesses. Researchers confirmed that the prevalence of diarrhea was 7% in ODF villages compared to 38% in OD villages [12]. Similar studies in Ethiopia also confirmed that the prevalence of under-five childhood diarrheal disease ranges from 9.9 to 17.2% in ODF villages and ranging from 23.2 to 36.3% in OD villages. Based on the WHO estimates, diarrhea contributes to more than one in every ten (13%) child deaths in Ethiopia [6, 13–15]. Moreover, open defecation also contributes significantly to the prevalence of neglected tropical diseases in Ethiopia, including intestinal worms, schistosomiasis, and trachoma. For example, some researchers reported that children were between 2.7 and 7.53 times at higher risk of active trachoma when they lived in OD prevalent households. These children will be blind as a result of repeated episodes of infection [16–18]. Therefore, the increment of open defecation will aggravate morbidity and mortality, particularly in children, which in turn affects the country’s socio-economic development.

Recently, increasing open defecation in Ethiopia was also due to open defecation-free slippage and high population growth. Most of Ethiopia’s ODF villages slipped back between 1 and 2 years of ODF certification [19]. Studies conducted regarding open defecation-free slippage rates in different parts of the country have shown varying results and conclusions. These studies reported a slippage rate between 7 and 28.4%. The indicators used to measure open defecation-free slippage and associated factors for slippage were also varied [2, 20]. Furthermore, a comprehensive study showing the country’s open defecation-free slippage rate with contributing factors was unavailable to guide policy reform and health planning.

So, this review aimed primarily to conduct a systematic review of the available literature of open defecation-free slippage rates in the country and to provide consolidated data showing the level of slippage and its associated factors. This review will provide comprehensive information contributing to policy reform on sanitation and hygiene-related health planning. It will also serve as baseline data for further study.

## Methods

### Review approach

We have employed a systematic review to estimate the pooled open defecation-free slippage rate and to determine the triggering factors of ODF slippage in Ethiopia. A mixed-method approach was used in this systematic review to assimilate data that have a qualitative nature (verbatim quotes and views) and quantitative data, whereas meta-analysis was also used to pool and present the data with a quantitative nature obtained from various studies [21]. The mixed-method is an integrative review, synthesizing the quantitative and qualitative data together [22]. The findings from this review were reported using the Preferred Reporting Items for Systematic Reviews and Meta-Analyses (PRISMA) tool or checklist (see S1 Table A) [23]. This systematic review was registered and available at PROSPERO ID CRD42020146950.

### Searching strategies

Systematic reviews containing registered protocols were primarily explored to avoid duplication. We have also systematically explored articles and grey literature from international databases including Cochrane Library, PubMed, Google Scholar, and Science direct. The following search terms “Open Defecation OR Open Defecation-Free OR Open Defecation-Free Slippage OR Community-Led Total Sanitation AND Ethiopia” were used separately and along with the Boolean operators like “OR” or “AND” to get published articles and grey literature. Our search was performed from December 1, 2013, to June 4, 2019 (see S2 Table B).

### Study inclusion criteria

We used the following inclusion criteria for screening of the studies: (a) articles dealing with open defecation, open defecation free, open defecation-free slippage, and Community-Led Total Sanitation; (b) all kinds of study designs; (c) articles in which the outcome measures were an open field defecating households in open defecation-free villages or ODF slippage rate (event rate); (d) article in which ODF is achieved only through CLTSH program or intervention; (e) articles in which determining factors for open defecation-free slippage were described; (f) published full-text articles and unpublished grey literature; (g) articles published in the English language; (h) studies conducted in Ethiopia; and (i) articles published between December 1, 2013, and June 4, 2019. Articles which did not meet the above criteria were excluded from the study.

### Data abstraction

Two researchers Thomas Ayalew Abebe (TAA) and Dr. Gudina Terefe Tucho (GTT) independently screened the titles and abstracts based on the inclusion criteria. They also removed duplicate articles. After removing duplicate articles, the full texts of the remaining articles were screened based on the inclusion criteria. The required information was extracted using a standardized data extraction format in Microsoft Excel and checked the data extraction process. These two researchers resolved their disagreements by consensus. The data extraction format included study characteristics such as the author name, region, study year, publication year, study design, study setting, sampling method, sample size, types of interventions to achieve ODF, ODF certification period, and open defecation-free slippage rate (event rate) (Table 1).

**Table 1:** Characteristics of 12 included studies to estimate the pooled ODF slippage rate and identify associated factors in Ethiopia 2020

Authors	Region	Study setting	Study year	Publication year	Study design	Sampling method	Sample size (sampled HH)	Intervention program to achieve ODF	ODF certification (year)	OD HHs (event)	ODF slippage rate (event rate^a^)
Ayalew et al. [13]	Amhara	CB	2016	2018	CC	Multi-stage	235	CLTSH	2013	30	0.128
Belachew et al. [24]	Tigray	CB	2106	2018	C	Multi-stage	450	CLTSH	2015	67	0.149
Crocker et al._K1 [19]	Oromia	CB	2013	2016	DID+Q	Multi-stage	800	CLTSH	2013	109	0.136
Crocker et al._K2 [19]	SNNP	CB	2013	2016	DID+Q	Multi-stage	733	CLTSH	2013	144	0.196
Crocker et al._K5 [19]	SNNP	CB	2013	2016	DID+Q	Multi-stage	1033	CLTSH	2013	275	0.266
Crocker et al._K6 [19]	SNNP	CB	2013	2016	DID+Q	Multi-stage	867	CLTSH	2013	216	0.249
Hunachew [25]	SNNP	CB	2013	2019	C+Q	Multi-stage	833	CLTSH	2011	208	0.25
Mamo et al. [14]	Oromia	CB	2017	2018	CC+Q	Multi-stage	380	CLTSH	2015	47	0.124
Negasa et al. [20]	Oromia	CB	2013	2015	C	Multi-stage	423	CLTSH	2011	120	0.284
Tyndale-Biscoe et al._Jimma [2]	Oromia	CB	2012	2013	S+Q	Multi-phase	802	CLTSH	2008	83	0.103
Tyndale-Biscoe et al._Shebedino [2]	SNNP	CB	2012	2013	S+Q	Multi-phase	975	CLTSH	2007	68	0.07
Roba [26]	Harari	CB	2014	2017	C+Q	SRS	420	CLTSH	2013	46	0.11
Megersa et al. [15]	Oromia	CB	2017	2019	CC	Multi-stage	355	CLTSH	2015	70	0.197
Tesfaye et al. [27]	SNNP	CB	2015	2018	C	Multi-stage	735	CLTSH	2013	124	0.169
Thomas [28]	Oromia	CB	2015	2016	CC	Multi-stage	322	CLTSH	2013	54	0.168
Tulu et al. [29]	SNNP	CB	2015	2017	CC	Multi-stage	352	CLTSH	2013	28	0.08
Total							9715			1689	

*C* cross-sectional, *CC* comparative cross-sectional, *DID* difference-in-difference design, *S* survey, *Q* qualitative (focus group discussion and in-depth interview), *OD* open defecation, *ODF* open defecation-free, *CB* community-based, *SRS* simple random sampling, *CLTSH* Community-Led Total Sanitation and Hygiene, *HH* households

^a^ODF slippage rate (event rate) = $\frac{\mathbf{OD}\ \mathbf{HH}\mathbf{s}\ \mathbf{from}\ \mathbf{sample}\mathbf{d}\ \mathbf{HH}\ \mathbf{in}\ \mathbf{ODF}\ \mathbf{village}\mathbf{s}\ \left(\mathbf{event}\right)}{\mathbf{Total}\ \mathbf{sample}\ \mathbf{size}\ \mathbf{from}\ \mathbf{ODF}\ \mathbf{village}\ \left(\mathbf{sampled}\ \mathbf{HH}\mathbf{s}\right)}$

### Quality assessment

To examine the risk of bias or methodological quality, we analyzed each article by using Hoy et al. [30] tool for prevalence studies. This instrument has 10 items used in two dimensions to evaluate the quality of studies: external validity (items 1–4: target population, sampling frame, sampling method, and non-response bias minimal) and internal validity (items 5–9: the data collection method, case definition, study instrument, and mode of data collection). Item 10 measures assessment-related bias. We also used a JBI Critical Appraisal Checklist for qualitative studies [31]. Two independent reviewers (TAA and GTT) critically assessed every article and addressed it. Finally, studies that scored low-risk bias were included in the systematic review (see S2 Table C and D).

### Data analysis

The extracted data were entered in Microsoft Excel format and analyzed using the Comprehensive Meta-Analysis version 3 statistical software. The heterogeneity was detected by reviewing the tables describing the included studies, reviewing the forest plots, and reviewing statistical tests. Heterogeneity in this review refers to the percentage of variation across studies. It was quantified using the inverse variance (*I*^2^). The *I*^2^ ranges from 0 to 100%. Hence, one rule of thumb categorized the *I*^2^ values of 25%, 50%, and 75% as low, moderate, and high heterogeneity, respectively, with a *p* value less than 0.05 [32].

Besides, the random effect meta-analysis model was employed for approximating the Der Simonian and Laird’s pooled effect [33]. The result was presented in the forest plot with respective event rates and 95% confidence intervals. Subgroup analysis was also performed among regions, sample size, and study design to identify the source of heterogeneity. The logit-transformed data were used for multivariate meta-regression for further investigation of heterogeneity and to appreciate the proportion of individual moderator’s effect on the heterogeneity between groups. Publication bias was detected with a visual inspection of funnel plots. Besides, Egger’s test was used to test the statistical significant asymmetry of the funnel plot’s statistical significant asymmetry, in which *p* < 0.05 was considered significant [34]. To account for any publication bias, we used the trim-and-fill method to correct the funnel plot and adjust the pooled estimate of the ODF slippage rate or event rate. The technique is known as “trim and fill” as the method initially trims the asymmetric studies from the right-hand side or left-hand side to locate the unbiased effect (in an iterative procedure) and then fills the plot by re-inserting the trimmed studies on the right or on the left as well as their imputed counterparts to the left or the right of the mean effect. If this shift is trivial, one can have more confidence that the reported outcome is valid [35]. Finally, we conducted a sensitivity analysis to investigate whether the pooled prevalence estimates were influenced even by a single study [36], whereas ODF slippage determining factors from quantitative studies and verbatim quotes or views regarding ODF slippage from qualitative studies not be pooled together with the use of the conventional meta-analytic technique [37]. To assimilate findings and to realize a more in-depth understanding of all factors that caused open defecation-free slippage, the researchers have followed the triangulation protocol [38]. Traditionally, quantitative methods were used to describe relationships between outcomes, while qualitative methods were applied to reveal the causes and intentions. However, the complicated open defecation-free slippage factors can only be identified by combining qualitative and quantitative methods, as presented in the mixed-method approach. This methodological approach (mixed-methods approach) incorporates qualitative data along with quantitative synthesis, which is a promising way of handling or negotiating heterogeneity [39].

This approach sets out to identify meta-themes across the studies from different methods, looking specifically at an agreement, partial agreement, or dissonance between these study findings. The mixed-method approach has been used in an Excel spreadsheet, contrasting themes from the qualitative and quantitative components, focusing specifically on inter-method discrepancies. Besides the previous methods, these integrated themes were presented following the structure of the quantitative analysis. The rate of open defecation-free slippage and the identified factors for open defecation-free slippage were the main findings of this systematic review.

## Results

### Study selection and characteristics of included studies

**Fig. 2:**
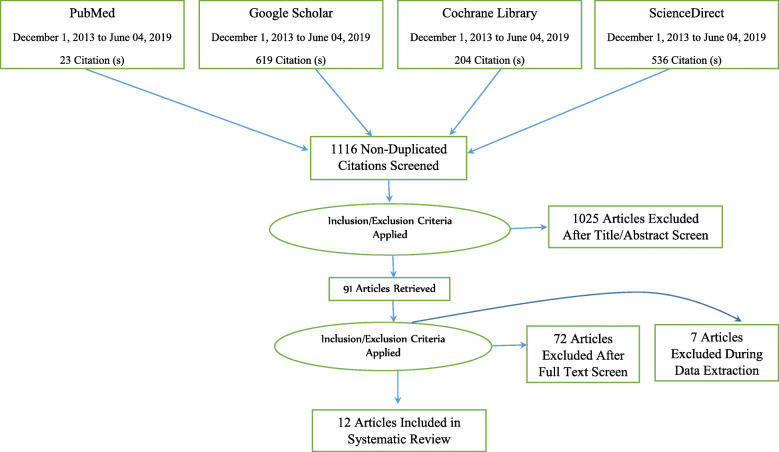
PRISMA flow diagram of included studies considered for the systematic review

A total of 1382 studies were identified from international databases and grey literature. We excluded 266 duplicate articles after reviewing the titles and abstracts. Then, after reading the title and the abstract based on preset inclusion criteria, 1025 studies were omitted. Finally, 91 studies were screened for full-text review, and 12 articles were selected to estimate the pooled open defecation-free slippage rate and associated factor in Ethiopia. The remaining 79 studies were excluded because they did not fulfill the pre-defined inclusion criteria (Fig. 2).

We considered two articles [Tyndale-Biscoe et al. [2] and Crocker et al. [19]] as six studies because they included the slippage rate of 6 study areas (2 from the Oromia region and four from the SNNP^1^ region). We segregated these two studies rather than merging the findings to minimize biased generalization during pooling the open defecation-free slippage rates. This systematic review included 9715 households, five Ethiopian administrative regions, and four study designs, sample sizes ranging between 235 and 975. The open defecation-free slippage rate also varied across the districts. Shebedino district in the SNNP region showed the lowest slippage rate (7%), whereas Kersa district in Oromia region showed the highest slippage rate (28.4%) (Table 1).

### Pooled Open defecation-free slippage rate

The result from the forest plot (meta-view) showed that 12 studies favored the risk of open defecation free slippage, whereas none of the studies favored ODF sustainability. If the event rate lies to the right of zero, it indicates open defecation-free slippage, and if it lies on zero, it shows open defecation-free status. However, here, zero has nothing to do with no effect. It only signifies no open defecation-free slippage or open defecation-free status. The data of open defecation-free slippage do not lie to the left side of zero because there was no negative effect size in this area of study. Open defecation free slippage data always skewed to the right or aligned on the centerline.

The overall result from the forest plot (meta-view) favored open defecation-free slippage because the red diamond fell to the right of the centerline. The red diamond represents a summary of the pooled open defecation free slippage rate of the studies.

**Fig. 3:**
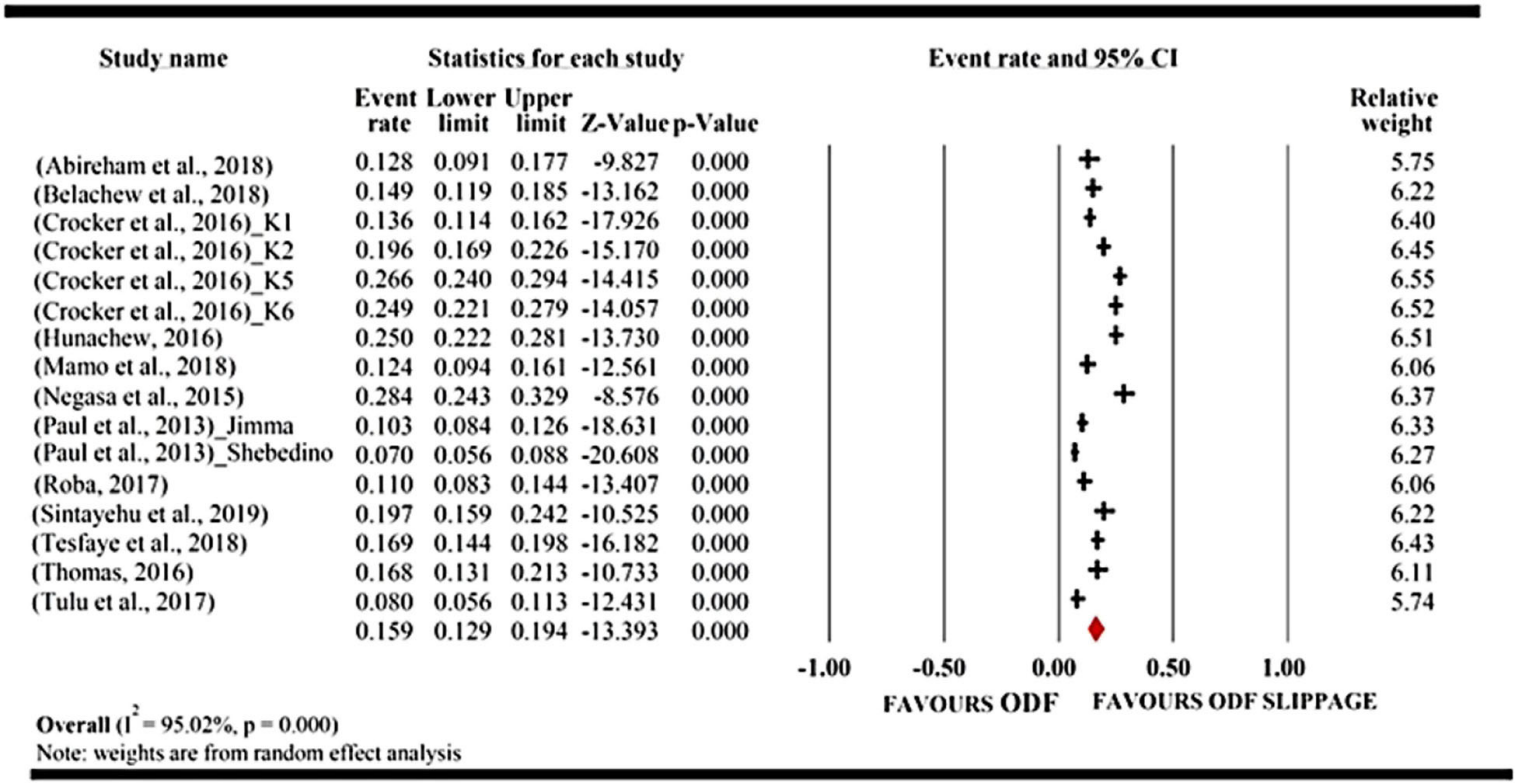
Forest plot of the pooled open defecation-free slippage rate in Ethiopia: meta-analysis

Hence, the results of 12 included studies in the meta-analysis showed that the pooled open defecation-free slippage rate in Ethiopia was 15.9% (95% CI 12.9–19.4%). Heterogeneity was also high (*I*^2^ = 95%; *P* < 0.0001). Therefore, the researchers used random-effects meta-analysis for controlling heterogeneity. The significant magnitude of heterogeneity also suggested conducting subgroup analysis to identify the source (Fig. 3).

### Publication bias assessment

On visual inspection, the funnel plot was found to be asymmetric (see S3 Figure A), and Egger’s test of the intercept (*B*_0_) came to be − 10.76 (95% CI − 16.80, − 4.71; *P* = 0.002). This suggested the existence of publication bias that requires the trim-and-fill method to adjust the final pooled effect size. However, the trim-and-fill techniques indicated no studies were missing, and ODF slippage rate was unchanged based on a random-effects model (see S3 Figure B and C). According to the sensitivity test, we found the source of Funnel plot asymmetry was due to the segregation of two studies [Tyndale-Biscoe et al. and Crocker et al.] for the issues of generalization. But when we merged the results of these studies, the pooled ODF slippage rate was decreased to 15.6% and heterogeneity between studies increased to 95.5%, whereas the finding from the egger test showed statistically insignificant (*P* = 0.11) with the intercept (*B*_0_) of − 4.66 (95% CI − 10.52, 1.21). Therefore, the reported ODF slippage rate (15.9%) is valid, and funnel plot asymmetry did not arise from publication bias.

### Subgroup analysis and meta-regression

We accompanied a subgroup analysis based on the study area, sample size, and study design (Table 2). When limiting our analysis to the study area, SNNP region showed the highest open defecation-free slippage rate of 17% (95% CI 12.3–23%) followed by the Oromia region with pooled open defecation-free slippage rate of 16.1% (95% CI 11.6–22.1%), but not statistically significant (*p* = 0.215) difference.

**Table 2:** Subgroup analysis of open defecation-free slippage rate in Ethiopia, 2020

Subgroup	Number of studies	Statistics for each study based on random model					
		Event rate (%)	Lower limit (%)	Upper limit (%)	Heterogeneity across the studies		Heterogeneity between the studies
					*I*^2^	*p* value	*p* value
**Region**							
Amhara	01	12.8	9.1	17.7	0.00	1.00	0.215
Harari	01	11.0	8.3	14.4	0.0	1.00	
Oromia	06	16.1	11.6	22.1	93.34	0.000	
SNNP	07	17.0	12.3	23.0	96.61	0.000	
Tigray	01	14.9	11.9	18.5	0.00	1.00	
**Sample size**							
> 500	08	16.6	15.3	18.1	91.10	0.000	0.546
< 500	08	19.4	18.4	20.4	96.72	0.000	
**Study design**							
Comparative cross-sectional	05	14.5	12.9	16.3	82.25	0.000	**< 0.001**
Cross-sectional	05	20.5	19.0	22.1	93.64	0.000	
Difference-in-difference	04	22.2	20.8	23.6	94.15	0.000	
Survey	02	8.6	7.4	10.0	83.60	0.000	

When restricting our analysis based on sample size (< 500 and ≥ 500), the sample size ≥ 500 subgroup had a higher open defecation-free slippage rate of 16.9% (95% CI 12.5–22.4%), whereas sample size < 500 had 14.8% (95% CI 11.0–19.7%); however, the difference between the groups was not statistically significant (*p* = 0.546).

Finally, when restricting our analysis by study design, the open defecation-free slippage rate was greater in the difference-in-difference design (25.40%) and lower in the survey (8.6%). In addition, heterogeneity between studies was statistically significant (*p* value < 0.001).

The presence of statistically significant heterogeneity from univariate subgroup analysis on the study design motivated us to conduct meta-regression, Thus, we found that only study design has a statistically significant heterogeneity with *Q* = 13.48 and *p* = 0.0037. Hence, the study design explained 37% (*R*^2^ analog = 0.37) of the heterogeneity between studies. The remaining unexplained heterogeneity was from the data (see S2 Table E).

### Pooled factors for open defecation-free slippage

**Fig. 4:**
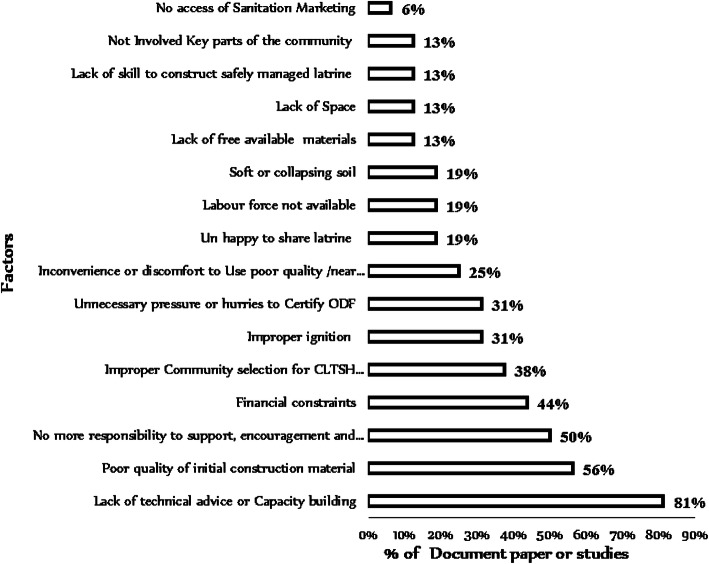
Factors for open defecation-free slippage in Ethiopia, 2020

The results in Fig. 4 show the summary of the analyzed factors for open defecation-free slippage from quantitative and qualitative studies using a mixed-method approach. The most appeared theme in 13 studies or 81% of research documents was the lack of technical advice or capacity building as the leading cause of open defecation-free slippage, while the least appeared theme in 6% study was inaccessible sanitation marketing.

## Discussion

### Open defecation-free slippage rate

**Fig. 5:**
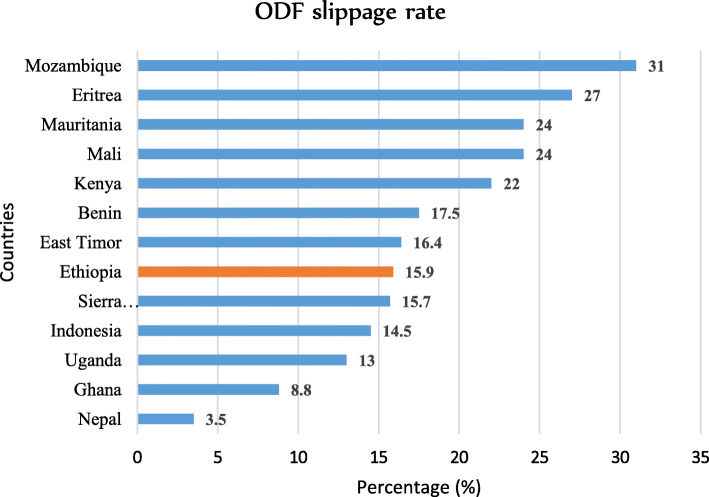
Distribution of open defecation-free slippage rate in some countries, 2020

The analysis revealed that the open defecation-free slippage rate in Ethiopia was 15.9% (95% CI 12.9–19.4%). This slippage rate was higher than a study conducted in Nepal 3.5% [40], in Ghana 8.8% [41], and in Indonesia 14.5% [42], consecutively. Similarly, the slippage rate was higher than the average slippage rate of some African countries, which was 10–13% [2, 43], in contrast, lower than East Timor 16.4% [44], Benin 17.5%, Mali and Mauritania 24% [41], Kenya 22% [2], Eritrea 27% [45], and Mozambique 31% [46] (Fig. 5). The reasons for open defecation-free slippage rate differences were implementation frameworks, socioeconomic status, and study design.

### Factors contributing to open defecation-free slippage

The researchers of this review identified many factors for open defecation-free slippage that have emerged from qualitative content analysis and quantitative assessments and grouped these factors as socio-economic, technical, and policy-related issues.

#### Socioeconomic issues

##### Discomfort to use shared latrines

Nineteen percent of the selected studies indicated shared toilets as a contributing factor for open defecation-free slippage. Studies conducted in 13 African countries also confirmed that sharing latrines brought frustration even among family members. The poorest people without option and sharing toilet from their neighbors slipped back to open defecation [47]. However, regular latrine sharers were relatives of latrine owners or the same family members or latrine co-investors. Sharers feel extreme embarrassment of asking to use the toilet regularly due to social taboos that prohibited them. Sharers feel ashamed of sharing the toilet with owners and subjected to a long waiting line (queue), which discouraged them from regular utilization of shared latrines [48]. A study report from Kenya similarly showed that sharing latrine with neighbors has a significant contribution for open defecation-free slippage [49]. Therefore, sharers remain or continue defecating in open field until they own private latrine.

##### Financial constraints

Seven (44%) of the research documents revealed that the reason for open defecation-free slippage was lack of money for maintenance. For this reason, they slipped back to open defecation or stopped using their latrines [50]. Most poor households cannot sustain open defecation-free status or rebuild a high-quality toilet (which stays longer) without financial support [43].

##### Lack of space

Some studies reported it as a contributing factor for open defecation. Households with filled latrines start open defecation because of inadequate space for reconstruction [4]. A similar report in Zambia revealed that the reason behind open defecation-free slippage was land constraint or inability to manage filled latrines [51]. Limited availability of land, especially among disadvantaged people, leads to continued open defecation practice [44, 52, 53]. Households with space or land can easily re-construct their latrine with any available materials [40].

##### Lack of freely available materials

Thirteen percent (13%) of the reviewed studies showed a lack of freely available materials which was a barrier for sustaining open defecation-free status. The impact was high among poor people with limited free access to durable materials. Thus, latrine construction using short-lasting materials results in low-quality latrine and elevated open defecation-free slippage [52]. A survey from Katete District Eastern Zambia indicated that people must travel approximately 10 km to get freely accessible materials used for constructing or maintaining latrines [54]. The subsidies for freely available materials in Indonesia empowered poor communities to build their toilet [55]. Thus, locally available materials can strengthen the sustainability of open defecation-free status through acceptable subsidies. Otherwise, the use of low-quality materials for constructing a latrine basement and supper structure will facilitate to an easily collapsible and impermanent toilet, which triggers open defecation. For instance, using defective construction materials such as mud or grass will lead to frequent latrine collapse and open defecation practices [56].

#### Technical issues

##### Lack of technical support

Thirteen (81%) research documents reported a lack of technical support as contributing factors for open defecation-free slippage. The evaluation of Community-Led Total Sanitation and Hygiene effectiveness in 8 African countries similarly stated that interventions designed to advise on upgrading and improving sanitation facilities using local materials maintain open defecation-free status [57]. Sanitation programs should include information and advice on various low-cost toilet designs and options [43]. If communities wonder how to create and express their need for sustainable latrines, the facilitators of CLTSH should provide technical advice [4].

##### Lack of skill

Lack of skill to construct safely managed latrine is another underlying factor for open defecation-free slippage. Usually, unskilled persons build a highly collapsible toilet. Latrine construction skills and technical advice help in the selection of better locally available materials and the correct latrine design. The unskilled person is unable to innovate good-quality latrine design. The few innovators available in the village were also unwilling to share their skills for others. Thus, most of the existing toilets were identical and unsanitary [58]. People built what they saw in their neighbors. For instance, a report from the Houaphanh Province of Lao showed that most of the toilets were 1 meter or less deep, with floors and walls constructed of temporary wooden poles or bamboo and a squatting hole with no lid cover, and many toilets were dirty [59]. Research in Kenya also shows that people copied most of the latrine prototypes from previously built toilets with limited knowledge based on the advice of their leaders. These lower-quality latrines had no privacy, unpleasant smell, and technical faults. Some common technical errors were the depth of the pit, shape, and absence of a lid on a slab hole [43]. Thus, the absence of practical guidance and lack of skilled labor in the community frequently results in low-quality unhygienic toilets and the preference of open defecation [44, 48, 60].

##### Poor quality of toilet

Poor quality of toilet was one of the contributing factors for open defecation-free slippage, as reported in 25% of the analyzed research. Poor-quality latrine pushes back users because of unaesthetic conditions (unpleasant smell or no privacy). Similarly, a study conducted in seven developing countries reported poor-quality latrine was a primary challenge for the sustainable use of a toilet in open defecation-free villages [61]. Supporting a study conducted in Kampala, Uganda, also showed that owning dirty and malfunction toilets descended the sanitation ladder back to open defecation because people were scared of falling in this toilet or gaining the disease from it [62]. Another study in India indicated that the construction of low-cost temporary latrine contributes to the slippage of open defecation-free practice [63]. A high-quality toilet can sustain open defecation-free status for a prolonged time, while a short-lived latrine discourages individuals from using or preferring open defecation [43].

##### Soft collapsible soil

Soft collapsible soil was another contributing factor for frequent latrine collapse and open defecation. The study conducted in Mozambique illustrated that 60% of people did not rebuild their latrine in sandy soil [64]. Latrine construction on sandy soil will have only a few months of life [65, 66]. People who built their toilet on sandy soil fell back to open defecation because of latrine collapse [67, 68].

#### Policy issues

##### Improper ignition or triggering

Improper ignition or triggering theme frequently appeared in five analyzed papers (*n* = 5, 31%). Improper CLTSH triggering drove people to open defecation-free slippage. So, attention for the three elements of CLTS triggering (shame, disgust, and fear) can have a long-lasting effect on sustained hygienic behavior [4]. A study report from 80 CLTSH triggered villages of East Java showed a significant relationship between better quality triggering and sustained open defecation-free status [50, 60, 69].

##### Early certification of ODF

Early certification of ODF appeared in 31% (*n* = 5) of the analyzed documents. The unacceptable pressure on communities for early certification of ODF will contribute to slippage. Technology acceptability and changing behavior toward its utilization require a sufficient time of adoption. The ODF certification based on social pressure and solidarity has a long-term effect on ODF sustainability [4, 70]. The exertion of tension by political leaders to declare open defecation-free ends up with unreal ODF status, while the communities were defecating in the open field [71]. Therefore, programmers should understand the reality of losing established facilities or preference of habitual behavior under tense pressure of implementation [72].

##### Lack of involvement

In 16% (*n* = 2) of the research papers, the lack of enrollment comprehends all segments of the population in the sanitation system led to ODF slippage. Most programs ignored engagement, particularly disadvantaged people such as needy families, elders, children, disabled persons, women, and other vulnerable community members. Ignoring these groups of people affects the sustainable performance of open defecation-free status. An evaluation report in Sierra Leone depicted that children who continued open defecation were students who went to school exactly at the same time as CLTSH triggering or who did not attend the CLTSH triggering event [73]. Another study conducted in 6 regions of Eritrea concluded that people who missed attending an ignition or CLTSH triggering moment could not stop open defecation [45]. Villages with more robust community involvement also have a low open defecation-free slippage rate [42]. A research report from Abidjan, Ivory Coast, also confirmed that “the effective sanitation interventions involve women and religious groups” [74]. The involvement of these groups is a critical stage for open defecation-free sustainability. Poor and disadvantaged households are at a much higher risk of open defecation-free slippage. Furthermore, they have a higher gap in sanitation and hygiene services because of equity and inclusion issues. The roles of women, adolescent girls, and boys need harmonization and integration in WASH programs. For example, women are more active and motivated than men to stop open defecation due to women shoulder most of the household activities [43, 48, 55, 75]. Therefore, the best way to open defecation-free sustainability relies on empowerment, equity, inclusion, consultation, and consideration of marginalized people in policy or program implementation.

### Policy implications

The 2030 sustainable development agenda comprises 17 goals and 169 targets. It included all nations across the world. In 2015, these countries introduced SDGs to end poverty and hunger, to protect the planet from destruction, to ensure prosperous life, to promote stability, and to build alliances. The SDGs’ strategic implementation target will be achieved by integrating the economic, social, and environmental aspects together over the next decade in areas of critical importance to humanity and the planet [76]. However, recent open defecation-free slippage is a severe obstacle to the achievement of the SDG target, in particular SDG 1 and SDGs 3–6.

#### How does ODF slippage affect SDG?

##### Eradication of poverty (SDG 1)

Our world loses 7 billion USD per year for health services because of sanitation-related diseases [77]. Open defecation is associated with child health and growth, which results in stunting [78, 79]. Stunting diminishes educational and productivity outcomes [50, 80, 81]. Recently released econometric reports have revealed that open defecation affects the national economy [79, 82, 83]. For instance, Kenya loses $88 million per year because of open defecation-related health impact. They spent this money on health care, medicines, and treatment. Correspondingly, open defecation-related diseases result in the loss of several working days [80]. It requires further research to know the econometric relationship between health and ODF slippage.

##### Creation of a healthy society (SDG 3)

Scientists noted poor sanitation as the cause of morbidity and mortality for the first time in 1842 and considered it as “great sanitary awakening” [84]. Open defecation is the most dangerous poor sanitation practice because 1 g of fresh feces carries 10^6^ viruses, 10^6–8^ bacteria, 10^4^ protozoan cysts, and 10^1–4^ helminth eggs [85]. Open defecation also plays a significant role in the transmission of infectious diseases; neglected tropical diseases and malnutrition, such as diarrhea, cholera, typhoid, shigellosis, hepatitis, helminths, trachoma, schistosomiasis, or bilharziasis; stunting; growth retardation; and anemia [86–90]. For example, the outbreak of cholera is associated with drinking water contaminated with feces [91]. The diarrheal disease kills more than 1.6 million under-five children every year, mainly because of poor sanitation [92]. Soil-transmitted helminths spread and re-infects in an open defecation-free slipped environment because ovas of helminths live in soil up to 2 years [93, 94]. Open defecation-free slippage also leads to the spread of trachoma—the leading cause of blindness [28, 87, 95]. Besides, intestinal schistosomiasis is prevalent in countries where open defecation is endemic [96–100]. Another common waterborne disease related to open defecation-free slippage is hepatitis (hepatitis A and hepatitis E). The occurrence of hepatitis is through contamination of drinking water sources with feces. Particularly, hepatitis E (emerging pathogen) will be dangerous for the life of pregnant women in open defecation-free slipped villages [101, 102].

##### Bringing inclusive, fair, and high-quality learning environments (SDG 4)

Open defecation practice cannot ensure privacy, dignity, safety, user-friendly, and a complete learning environment. Exposing school children to open defecation leads to an increased risk of respiratory, gastrointestinal, neurocognitive, and psychological illnesses [103–105]. The lack of safe sanitation services in schools also increases school absenteeism, especially for girl students [77, 103, 106–108]. School absenteeism can lead to low-grade achievement (low academic performance), failure to pass classes, increase drop-out rates, and delays in social development [106, 109–111], which restrains children from gaining economic and health benefits related to educational achievement [112].

##### Ending open defecation by 2030 (SDG 6)

Ending open defecation by 2030 (SDG 6) will be difficult because of open defecation-free slippage. The planet has reduced open defecation radically since 2000, except for sub-Saharan Africa and Oceania. WHO and UNICEF reported that the factor for increment in open defecation was high population growth. However, the researchers of this review argue that population growth was not the only reason for open defecation increment, but the occurrence of open defecation-free slippage was another factor for the increment [1]. If ODF slippage continues, ending open defecation by 2030 will be fictitious altogether with the achievement of universal sanitation access and ascent to the sanitation ladder.

#### The implication of ODF slippage in Ethiopia

Ethiopia is one of the top-piloted countries where open defecation tremendously decreased between the years 2000 and 2015. Even though the country has many most effective enabling environments, the reality depicts behavior change, and the implementation process through total sanitation and hygiene program is in back-and-forth progress because of emerging ODF slippage.

Open defecation-free slippage will be an obstacle for achieving the Ethiopian Health Sector Development Plan (HSDP) in decreasing the percentage of households using latrines and the proportion of villages (kebeles) free of open defecation [113]. Moreover, open defecation-free slippage leads to the failure of the One WASH National Program (OWNP), which intended to achieve three primary hygiene and sanitation behaviors such as use safely managed excreta, apply a safe water chain from a safe source to the mouth, and wash hands with soap or a substitute after defecation [114]. It will also affect the performance of the Ethiopian Second Growth and Transformation Plan (GTP). GTP II focused on tripling improved household latrine coverage from 28% in 2015 to 82% by 2020, as well as doubling the ODF kebeles from 27 to 50% by the end of the 2018/2019 budget year, but the WASH sector extended this period to 2024 due to deadline [115, 116]. The challenge goes to the success of the Ethiopian National Hygiene and Sanitation Strategy (NHSS) that basis on new thinking and effort to shift the sluggish annual growth rate (1.2%) of improved household latrine to reach 51% by the end of 2020. NHSS does not safeguard the environment and society unless this ODF slippage ceases to exist with the introduction of innovative ideas to stop slippage. Besides, it would take another 25 years to achieve 51% of improved sanitation facilities [117].

The prevalence of waterborne diseases and neglected tropical diseases (especially soil-borne helminths, trachoma, and schistosomiasis) is increasing in Ethiopia due to open defecation-free slippage. These diseases increase maternal and childhood morbidity and mortality, which in turn impacts the economic development of the country [15, 118–120].

### Alternative sanitation policy

Policy modification for equity and inclusion of poor and marginalized groups contributes to greater ODF sustainability. Therefore, it requires prioritization of the following activities at different levels.

At a technical level, it is crucial to identify the best hygiene promotion approach and affordable sanitation technology and determine the minimum cost that will not discriminate the poor and marginalized groups. The strengthening of best approaches such as Community-Led Total Sanitation and Hygiene program and school WASH program will achieve sustained open defecation-free communities. The program like post-open defecation-free follow-up should be incorporated in the National Sanitation Program.

At the government or donor level, the strategies and policies should better incorporate regular follow-up with measurable indicators. For example, including post-open defecation-free follow-up in the Community-Led Total Sanitation and Hygiene program will sustain the learned hygienic behavior. The government or donor should better consider the disadvantaged people who lack the resources (land or space, money, and local material) with acceptable subsidy programs because it will help them climb the sanitation ladder from a shared toilet to a private toilet or from a simple pit latrine to an improved sanitation facility. The increment of budget allocation for sanitation and hygiene programs also has a significant impact on program sustainability [42].

At the researchers’ level, it is necessary to identify and test improved sanitation technology and estimate their cost. For instance, strategies that integrate Community-Led Total Sanitation and Hygiene with other low-cost technologies like ecological sanitation (biogas, Arboloo, urine-diverting dry toilets, and a twin pit) have a significant impact on open defecation-free sustainability. It is common for rural communities to dig new pit after filled latrines, and finally, no reconstruction space or land left because of no tradition of reusing the pit. Furthermore, they have no idea that human excrement is used as a source of energy and organic fertilizer.

Our findings indicate that applying these three alternatives helps eradicate open defecation or sustain hygienic behavior in the community. The existing National Sanitation and Hygiene strategy should include post-ODF program for the most straightforward implementation and for a better capacity to address ODF slippage. Finally, an appropriate sanitation technology selection, sanitation behavior remodeling, and assessing the levels of social belief help in sustaining open defecation-free status.

## Conclusion

Even though Ethiopia has adopted Sustainable Development Goal and reduced open defecation for decades, still the progress of improved sanitation facility is steady, and the ODF-certified villages were also retiring. It was estimated that one out of six Ethiopian households engaged in open defecation after they certified ODF status. Therefore, the increment of open defecation practice in Ethiopia was due to open defecation-free slippage besides population growth.

The factors for open defecation-free slippage were lack of technical support, poor-quality construction material, lack of follow-up, financial constraints, improper implementation of Community-Led Total Sanitation and Hygiene program, demotivation due to the poor-quality latrine, demotivation using shared latrines, collapsible soil conditions, lack of freely available materials, lack of land, lack of involvement, and lack of promotion on sanitation marketing.

Consequently, to accelerate the elimination of open defecation, the government of Ethiopia and its partners should better consider the level of slippage and its associated factor. Furthermore, there should be an integration of post-open defecation-free program and multi-sectoral coordination in the sanitation program. Finally, the encouragement of further research on pro-poor improved sanitation technology will help the community in climbing the sanitation ladder. Therefore, it will be easy to attain sustainable open defecation-free status or end open defecation by the end of 2030.

## Supplementary Information

**Additional file 1.** Table A. PRISMA 2009 Checklist for Open defecation free slippage assessment in Ethiopia, 2020.

**Additional file 2.** Supplementary tables B–E.

**Additional file 3.** Supplementary figures A–C.

## Data Availability

Data will be available upon request of the corresponding author.

## Abbreviations

CLTSH: Community-Led Total Sanitation and Hygiene
EDHS: Ethiopian Demographic and Health Survey
GTP: Growth Transformation Plan
HSDP: Health Sector Development Plan
NHSS: National Hygiene and Sanitation Strategy
OD: Open defecation
ODF: Open defecation-free
OWNP: One WASH National Program
PRISMA: Preferred Reporting of Systematic Reviews and Meta-Analysis
SDGs: Sustainable Development Goals
SNNP: Southern Nation and Nationalities People
UNICEF: United Nations International Children’s Emergency Fund
USD: United States dollar
WASH: Water, sanitation, and hygiene
WHO: World Health Organization

## Footnotes

1 South Nation, Nationalities and Peoples (SNNP)
