# Supplementary material for: Open defecation-free slippage and its associated factors in Ethiopia: a systematic review
Source: Syst Rev. 2020 Nov 3;9:252. doi: 10.1186/s13643-020-01511-6 (PMC7641843; doi:10.1186/s13643-020-01511-6)
Supplement: Supplementary file 2 — Additional file 2. Supplementary tables B–E. [file 13643_2020_1511_MOESM2_ESM.docx]

| Search engine | Terms and syntax | Search result in number hit | Duplication | | Screened based on Title and Abstract read | Retrieved Documents based on Full Text Articles Read and during Data Extraction |
| --- | --- | --- | --- | --- | --- | --- |
|  |  |  | Within  search  engine | Between  search engine |  |  |
| PubMed | (((Open Defecation) OR (Open Defecation Free)) OR (Open Defecation Free Slippage)) AND (Ethiopia)  *From 2013/12/1 - 2019/6/04* | 23 | 0 |  | 10 | Four (4) literature  (#1, #4, #5 and #7)  All were published literatures |
| Google Scholar | Open defecation Ethiopia "community led total sanitation"  From 2013/12/1 - 2019/6/04 | 619 | 93 |  | 58 | All of **12** literature  (On Page 1, five literatures at: #2, #3, #6, #9, and #13)  (On Page 2, three literatures at: #3, #8, and #12)  (On Page 3, two literatures at: #8, and #9)  (On Page 4, one literatures at: #17)  (On Page 9, one literatures at: #3)  Ten published literatures and 2 Grey literature  NB: the colors shows duplicate literature |
| Cochrane Library | open defecation in Title Abstract Keyword OR open defecation free in Title Abstract Keyword AND "Ethiopia" in Title Abstract Keyword  From 01/12/2013 - 04/06/2019 | 204 | 6 |  | 8 | 0 |
| ScienceDirect | "Open Defecation" OR "Open Defecation Free" OR "Open Defecation Free Slippage" AND "Ethiopia"  *From 2013/12/1 - 2019/6/04* | 536 | 0 |  | 15 | 0 |
| Total |  | 1382 | 99 | 147 | 91 | ***12*** |

**Table B**: Shows searching strategy for open defecation free slippage in Ethiopia, 2020.

**Table C**: Summary of Quality assessment tool of quantitative studies used to estimate ODF slippage rate in Ethiopia, 2020 (adapted from Hoy et al).

| Sr.No | Risk of bias items | INCLUDED STUDIES | | | | | | | | | | | |
| --- | --- | --- | --- | --- | --- | --- | --- | --- | --- | --- | --- | --- | --- |
|  |  | (Abireham et al., 2018) | (Belachew et al., 2018) | (Crocker et al., 2016)_K1 | (Hunachew, 2016) | (Mamo et al., 2018) | (Negasa et al., 2015) | (Paul et al., 2013)_Jimma | (Roba, 2017) | (Sintayehu et al., 2019) | (Tesfaye et al., 2018) | (Thomas, 2016) | (Tulu et al., 2017) |
| 1 | Was the study’s target population a close representation of the national population in relation to relevant variables, e.g. age, sex, occupation? (1,0) | 0 | 0 | 0 | 1 | 0 | 0 | 0 | 0 | 0 | 0 | 1 | 0 |
| 2 | Was the sampling frame a true or close representation of the target population? (1,0) | 0 | 0 | 0 | 0 | 0 | 0 | 0 | 0 | 0 | 0 | 0 | 0 |
| 3 | Was some form of random selection used to select the sample, OR, was a census undertaken? (1,0) | 0 | 0 | 1 | 0 | 0 | 0 | 0 | 0 | 0 | 0 | 1 | 0 |
| 4 | Was the likelihood of non-response bias minimal? (1,0) | 0 | 0 | 0 | 0 | 0 | 0 | 0 | 0 | 0 | 0 | 0 | 0 |
| 5 | Were data collected directly from the subjects (as opposed to a proxy)? (1,0) | 0 | 0 | 0 | 0 | 0 | 0 | 0 | 0 | 0 | 0 | 0 | 0 |
| 6 | Was an acceptable case definition used in the study? (1,0) | 1 | 1 | 1 | 0 | 1 | 1 | 0 | 1 | 1 | 1 | 1 | 1 |
| 7 | Was the study instrument that measured the parameter of interest (open defecation free slippage rate) shown to have reliability and validity (if necessary)? (1,0) | 0 | 0 | 0 | 0 | 0 | 0 | 0 | 0 | 0 | 0 | 1 | 0 |
| 8 | Was the same mode of data collection used for all subjects? (1,0) | 0 | 0 | 0 | 1 | 0 | 0 | 0 | 0 | 0 | 0 | 0 | 0 |
| 9 | Were the numerator(s) and denominato r(s) for the parameter of interest appropriate? (1,0) | 0 | 0 | 0 | 0 | 0 | 0 | 0 | 0 | 0 | 0 | 0 | 0 |
|  | Sum | 1 | 1 | 2 | 2 | 1 | 1 | 0 | 1 | 1 | 1 | 4 | 1 |
| 10 | Summary on the overall risk of study bias (LOW RISK = 0-3, MODERATE RISK 4-6, HIGH RISK 7-9) | Low | Low | Low | Low | Low | Low | Low | Low | Low | Low | Moderate | Low |

**Table D**: Summary of Quality assessment tool for qualitative studies used to identify ODF slippage factors in Ethiopia, 2020 (adapted from Lockwood et al).

| Sr.No | Risk of bias items | INCLUDED STUDIES | | | |  |
| --- | --- | --- | --- | --- | --- | --- |
|  |  | (Crocker et al., 2016) | (Hunachew, 2016) | (Mamo et al., 2018) | (Paul et al., 2013) | (Roba, 2017) |
| 1 | Is there congruity between the stated philosophical perspective and the research methodology? (1,0) | 1 | 1 | 1 | 1 | 1 |
| 2 | Is there congruity between the research methodology and the research question or objectives? (1,0) | 1 | 1 | 1 | 1 | 1 |
| 3 | Is there congruity between the research methodology and the methods used to collect data? (1,0) | 1 | 1 | 1 | 1 | 1 |
| 4 | Is there congruity between the research methodology and the representation and analysis of data? (1,0) | 1 | 1 | 1 | 1 | 1 |
| 5 | Is there congruity between the research methodology and the interpretation of results? (1,0) | 1 | 1 | 1 | 1 | 1 |
| 6 | Is there a statement locating the researcher culturally or theoretically? (1,0) | 0 | 0 | 1 | 1 | 1 |
| 7 | Is the influence of the researcher on the research, and vice- versa, addressed? (1,0) | 1 | 0 | 1 | 1 | 1 |
| 8 | Are participants, and their voices, adequately represented? (1,0) | 1 | 1 | 1 | 0 | 1 |
| 9 | Is the research ethical according to current criteria or, for recent studies, and is there evidence of ethical approval by an appropriate body? (1,0) | 0 | 0 | 1 | 0 | 0 |
| 10 | Do the conclusions drawn in the research report flow from the analysis, or interpretation, of the data? | 1 | 1 | 1 | 1 | 1 |
|  | Sum | 8 | 7 | 10 | 8 | 9 |
| 10 | Summary on the overall risk of study bias (HIGH RISK = 0-4, MODERATE RISK 5-7, LOW RISK 8-10) | Low | Moderate | Low | Low | Low |

**Table E**: Random effects-Meta-Regression of selected Covariate with Logit event rate of open defecation-free slippage rate in Ethiopia, 2020.

| **Set** | **Covariate** | **Coefficient** | **Standard Error** | **95%** | | **Z-value** | **2-sided P-value** | **VIF** | **Statistics Between** |
| --- | --- | --- | --- | --- | --- | --- | --- | --- | --- |
|  |  |  |  | **Lower** | **Upper** |  |  |  |  |
| sample size | Intercept | -1.34 | 0.61 | -2.54 | -0.14 | -2.19 | 0.03 | 34.64 | Q=0.27, df=1, p=0.6009 |
|  | (based 500): <500 | 0.30 | 0.58 | -0.83 | 1.44 | 0.52 | 0.60 | 7.82 |  |
| Study design | Comparative Cross-sectional | -0.88 | 0.47 | -1.80 | 0.03 | -1.89 | 0.06 | 4.19 | **Q=13.48, df=3, p=0.0037^***^** |
|  | DID | -0.03 | 0.36 | -0.73 | 0.68 | -0.07 | 0.94 | 2.31 |  |
|  | Survey | -1.09 | 0.44 | -1.94 | -0.24 | -2.50 | 0.01 | 1.94 |  |
| Region | Harari | -1.05 | 0.77 | -2.56 | 0.45 | -1.37 | 0.17 | 3.09 | Q=4.35, df=4, p=0.3611 |
|  | Oromia | 0.11 | 0.49 | -0.86 | 1.08 | 0.23 | 0.82 | 5.31 |  |
|  | SNNP | 0.00 | 0.54 | -1.06 | 1.05 | -0.01 | 0.99 | 6.69 |  |
|  | Tigray | -0.71 | 0.76 | -2.21 | 0.79 | -0.92 | 0.36 | 3.16 |  |
| **Statistics for Model 1**  **Test of the model: Simultaneous test that all coefficients (excluding intercept) are zero**  Q = 15.18, df = 8, p = 0.0557  **Goodness of fit: Test that unexplained variance is zero**  Tau² = 0.1557, Tau = 0.3946, I² = 92.26%, Q = 90.44, df = 7, p = 0.0000 | | | | **Comparison of Model 1 with the null model**  **Total between-study variance (intercept only)**  Tau² = 0.2393, Tau = 0.4892, I² = 95.02%, Q = 301.44, df = 15, p = 0.0000  **Proportion of total between-study variance explained by Model 1**  R² analog = 0.35 | | | | | |
