# Supplementary material for: Open defecation-free slippage and its associated factors in Ethiopia: a systematic review
Source: Syst Rev. 2020 Nov 3;9:252. doi: 10.1186/s13643-020-01511-6 (PMC7641843; doi:10.1186/s13643-020-01511-6)
Supplement: Supplementary file 3 — Additional file 3. Supplementary figures A–C. [file 13643_2020_1511_MOESM3_ESM.docx]

**** **Figure A**: **Funnel plot for Logit-event rate estimate SE for open defecation-free slippage in Ethiopia, 2020.**


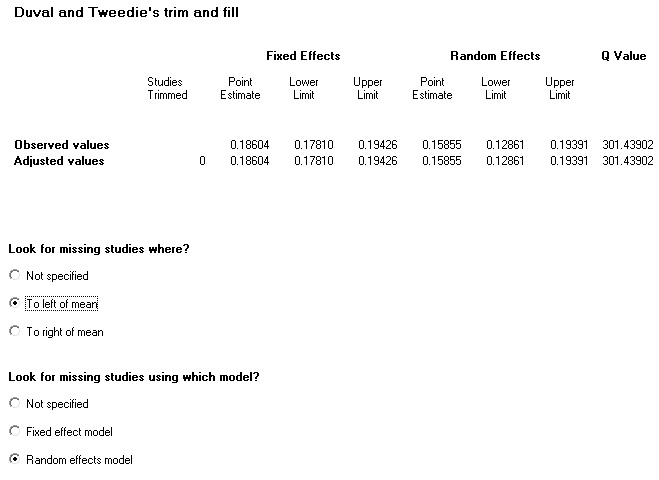


**Figure B: The trim and fill analysis to identify missing studies in open defecation-free slippage assessment in Ethiopia, 2020.**


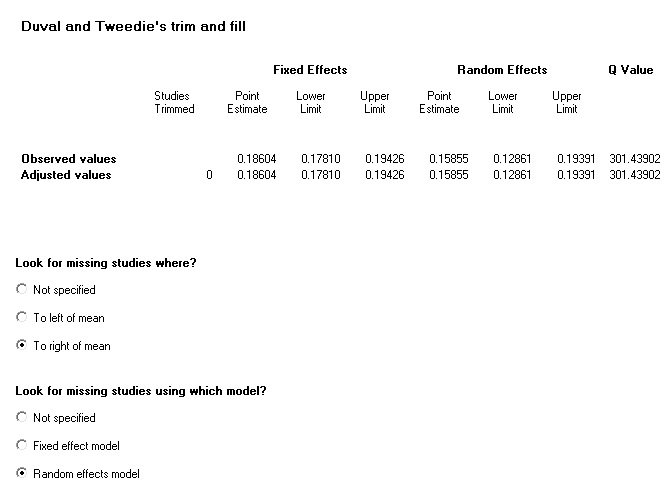


**Figure C: The trim and fill analysis to identify missing studies in open defecation-free slippage assessment in Ethiopia, 2020.**
